# Supplementary material for: A Rat Study to Evaluate the Protein Quality of Three Green Microalgal Species and the Impact of Mechanical Cell Wall Disruption
Source: Foods. 2020 Oct 24;9(11):1531. doi: 10.3390/foods9111531 (PMC7694116; doi:10.3390/foods9111531)
Supplement: Supplementary file 1 [file foods-09-01531-s001.pdf]

Table S1. Composition of experimental diets.

Ingredient[#]	Zero-protein	Casein	CV	CS	AO	CVMR	CSMR	AOMR
Ingredient (%)								
Casein	0	11.52						
CV			18.69					
CS				19.92				
AO					24.81			
CVMR						18.76		
CSMR							20.08	
AOMR								25.38
Corn starch	47.48	37.48	37.04	34.24	34.68	37.04	34.21	34.62
Sucrose	35	35	35	35	35	35	35	35
Lard[&]	9	9	4.25	4.78	3.54	4.23	4.74	3.42
Sunflower oil[δ]	1	1	1	1	1	1	1	1
Cellulose	2	2	1.23	1.60	0.68	1.23	1.60	0.65
Mineral mixture	4	3.63	3.01	3.04	3.03	3.01	3.04	3.01
Vitamin mixture	1	1	1	1	1	1	1	1
Choline bitartrate	0.2	0.2	0.2	0.2	0.2	0.2	0.2	0.2
Titanium oxide	0.3	0.3	0.3	0.3	0.3	0.3	0.3	0.3
Nutrient density (calculated, %)								
Energy (kcal/g)	4.27	4.22	4.19	4.22	4.09	4.19	4.22	4.08
Protein	0	9.89	9.83	9.89	9.59	9.83	9.88	9.56
Starch + sucrose	82.50	71.67	71.25	71.70	69.52	71.22	71.64	69.30
Fat	10.00	9.89	9.83	9.89	9.59	9.83	9.88	9.56
Fiber	2.00	1.98	1.97	1.98	1.92	1.97	1.98	1.91

[#] The ingredients were purchased from Dyets Inc. (Bethlehem, PA, USA) unless specified. [&] Tenderflake® Pure Lard containing 0.1g protein /10 g Lard (Walmart, Charlottetown, PE, Canada). [δ] Sunflower oil was purchased from the Atlantic Superstore (Charlottetown, PE, Canada). CV, CS, and AO are Chlorella vulgaris/NRC isolate AB12-A-U-BBM, Chlorella sorokiniana/NRC isolate SMC-15M and Acutodesmus obliquus/NRC isolate SMC-6F, respectively. CVMR, CSMR, and AOMR are the mechanically cell-ruptured CV, CS, and AO, respectively.

Table S2. Amino acid composition of casein and six microalgae products[1.]

AA (mg/g biomass)	Casein	CV	CS	AO	CVMR	CSMR	AOMR
Asparagine + aspartic acid	60.08	35.09	37.38	30.71	35.48	37.99	27.04
Glutamine + glutamic acid	191.34	42.24	45.93	35.16	39.41	45.39	29.27
Serine	51.65	18.19	18.25	20.70	18.99	18.23	19.05
glycine	15.56	20.80	23.00	19.22	22.46	23.86	18.93
histidine	20.11	6.33	7.44	4.32	6.52	7.20	3.55
arginine	32.27	31.41	26.94	17.81	32.06	28.66	15.61
threonine	36.22	15.34	19.20	17.94	16.20	18.41	17.37
alanine	25.28	30.09	30.34	28.50	32.27	30.98	28.26
proline	105.24	19.12	21.46	19.19	20.58	21.83	19.77
α-amino-N-butyric acid	0.14	0.36	0.47	0.73	0.31	0.51	0.61
tyrosine	46.92	15.83	15.83	12.94	15.78	16.17	11.40
valine	55.37	20.73	21.38	18.24	21.77	21.85	17.74
methionine	23.07	6.68	5.98	5.94	6.32	6.28	5.76
isoleucine	39.65	12.91	12.65	11.09	13.25	12.92	10.86
Leucine	74.63	32.84	30.51	26.14	33.75	30.64	25.33
phenylalanine	35.12	19.19	15.80	15.15	18.80	15.70	14.23
Ornithine	0.11	0.12	0.25	0.39	0.29	0.31	0.34
lysine	41.93	18.55	20.74	12.69	17.32	21.31	10.22
cysteine	2.21	3.64	3.91	4.15	2.89	4.16	3.92

tryptophan	13.28	8.31	7.15	6.04	8.34	8.30	6.40
ΣAA	870.2	358.9	364.9	312.5	364.1	371.0	290.5
N x 6.25[2]	868.5	535.0	502.1	403.4	533.3	497.8	394.6
N x 6.25 - ΣAA[3]	0	176.1	137.2	90.9	169.4	126.8	104.1

[1] Results are the average of 2 analytical replicates. [2] N x 6.25 is the total crude protein estimated by measuring total nitrogen using a nitrogen elemental analyzer. [3] The value of N x 6.25 - ΣAA for casein is -1.7 that was considered the analytical error and set to 0.
